# Exposure to arsenic in utero is associated with various types of DNA damage and micronuclei in newborns: a birth cohort study

**DOI:** 10.1186/s12940-019-0481-7

**Published:** 2019-06-07

**Authors:** Panida Navasumrit, Krittinee Chaisatra, Jeerawan Promvijit, Varabhorn Parnlob, Somchamai Waraprasit, Chalida Chompoobut, Ta Thi Binh, Doan Ngoc Hai, Nguyen Duy Bao, Nguyen Khac Hai, Kyoung-Woong Kim, Leona D. Samson, Joseph H. Graziano, Chulabhorn Mahidol, Mathuros Ruchirawat

**Affiliations:** 1 Laboratories of Environmental Toxicology/Chemical Carcinogenesis, Chulabhorn Research Institute, Laksi, Bangkok, 10210 Thailand; 2 Center of Excellence on Environmental Health and Toxicology, CHE, Ministry of Education, Ratchathewi, Bangkok, 10400 Thailand; 3 National Institute of Occupational and Environmental Health, Hanoi, Vietnam; 4 International Environmental Research Center, Gwangju Institute of Science and Technology, Gwangju, South Korea; 5 Center for Environmental Health Sciences, Massachusetts Institute of Technology, Cambridge, USA; 6 Department of Environmental Health Sciences, Columbia University, New York, USA

**Keywords:** Arsenic, Maternal exposure, In utero exposure, Genetic damage, 8-nitroguanine, 8- hydroxydeoxyguanosine, DNA strand breaks, Micronucleus

## Abstract

**Background:**

Growing evidence indicates that in utero arsenic exposures in humans may increase the risk of adverse health effects and development of diseases later in life. This study aimed to evaluate potential health risks of in utero arsenic exposure on genetic damage in newborns in relation to maternal arsenic exposure.

**Methods:**

A total of 205 pregnant women residing in arsenic-contaminated areas in Hanam province, Vietnam, were recruited. Prenatal arsenic exposure was determined by arsenic concentration in mother’s toenails and urine during pregnancy and in umbilical cord blood collected at delivery. Genetic damage in newborns was assessed by various biomarkers of early genetic effects including oxidative/nitrative DNA damage (8-hydroxydeoxyguanosine, 8-OHdG, and 8-nitroguanine), DNA strand breaks and micronuclei (MN) in cord blood.

**Results:**

Maternal arsenic exposure, measured by arsenic levels in toenails and urine, was significantly increased (*p* <  0.05) in subjects residing in areas with high levels of arsenic contamination in drinking water. Cord blood arsenic level was significantly increased in accordance with maternal arsenic exposure (*p* <  0.001). Arsenic exposure in utero is associated with genotoxic effects in newborns indicated as increased levels of 8-OHdG, 8-nitroguanine, DNA strand breaks and MN frequency in cord blood with increasing levels of maternal arsenic exposure. Maternal toenail arsenic level was significantly associated with all biomarkers of early genetic effects, while cord blood arsenic levels associated with DNA strand breaks and MN frequency.

**Conclusions:**

In utero arsenic exposure is associated with various types of genetic damage in newborns potentially contributing to the development of diseases, including cancer, later in life.

## Background

It has been estimated that 160–200 million people worldwide are exposed to levels of inorganic arsenic (iAs) in drinking water that exceed the World Health Organization (WHO) safety standard of 10 μg/L [1]. In Southeast Asia, groundwater, a primary source of drinking water, has been found to be a significant source of iAs exposure. Vietnam is one of the Southeast Asian countries facing serious public health problems from arsenic contamination of groundwater. The population at risk for chronic arsenic poisoning from exposure to arsenic-contaminated groundwater is approximately 10 million in Vietnam’s Red River delta [2] and 16 million in the Mekong Delta in Vietnam and Cambodia [3].

In Vietnam, one of the areas with the highest arsenic contamination is Hanam province, located 60 km from Southern Hanoi, where the level of arsenic in groundwater varied from 1 to 3050 μg/L [4]. Sand-filtered drinking water from various sources such as groundwater, piped water, and rainwater are commonly used in this area. More than 50% of the stored water, however, contained arsenic levels above the WHO recommended value [5]. Although skin cancer risk in adults due to consumption of filtered piped water was increased, the cancer risk would be 11.5 times higher if the water was not filtered [6].

In utero and early life exposures to arsenic in humans increase the risk of adverse health effects [7] and have been related to elevated risk of respiratory disease, cardiovascular disease, and cancer later in life [8]. Exposure to arsenic during pregnancy is of particular concern because it represents the period of vulnerability to toxicants for both mother and child. Fetal development represents an extremely rapid phase of organogenesis and growth, and is therefore highly sensitive to adverse effects of toxic exposures [9].

Evidence from human population studies suggests early life-stage sensitivity to iAs-induced carcinogenicity. Specifically, a population in Antofagasta, Chile, exposed to high levels of iAs in drinking water (0.87 ppm) in utero and/or during early childhood for a discrete period of 12 years had higher rates of lung, bladder, laryngeal, kidney and liver cancer mortality as adults, compared to other Chileans in the same age group [10]. Evidence obtained in mice also suggests that in utero exposure to iAs increases susceptibility to developing cancer later in life [11].

Arsenic-induced carcinogenesis involves the generation of reactive oxygen and nitrogen species (ROS/RNS) resulting in oxidative stress, which in turn induces genomic instability through DNA damage [12] and other genotoxic effects such as micronuclei (MN) formation and chromosome aberrations [13]. MN frequency in mononucleated cells provides an estimation of genome damage accumulated over a long period prior to blood sampling, whereas MN in binucleated cells represents the lesions that recently occurred. Arsenic exposure generates ROS during its biotransformation; trivalent arsenic is able to function as the main inducer of ROS generation within the cells [14]. In addition, ROS influences the activation of NF-κB, resulting in upregulation of inflammatory cytokines [15]. Activation of pro-inflammatory cytokines can trigger oxidant-generating enzymes, such as NADPH oxidase, myeloperoxidase, and inducible nitric oxide synthase, to produce ROS and RNS [16]. ROS and RNS induce the formation of mutagenic oxidative DNA lesions such as 8-oxo-7,8-dihydro-2′-deoxyguanosine (8-OHdG) and 8-nitroguanine, respectively. These are mutagenic lesions that preferentially lead to G:C to T:A transversion mutation [17]. Oxidative and nitrative stress, therefore, contribute to the damage of biomolecules, such as DNA, RNA, lipids, and proteins, leading to an increase in mutations, genomic instability, epigenetic changes, and protein dysfunction, and play roles in the carcinogenic process. 8-OHdG has been recognized as a good biomarker of oxidative DNA damage and 8-nitroguanine is associated with inflammation-related cancers such as cholangiocarcinoma [18, 19].

A recent study in a Thai cohort showed that arsenic exposure in utero increased levels of urinary 8-nitroguanine in newborns which significantly correlated with increased expression of inflammatory genes (*COX2, EGR1* and *SOCS3*) in cord blood [20]. A follow-up study showed that these arsenic-exposed children had increased urinary 8-nitroguanine [20] and salivary 8-OHdG as well as decreased expression of human 8-oxoguanine DNA glycosylase 1 (*hOGG1*), suggesting a defect in the repair of 8-OHdG [21]. These observations support the earlier findings in the same cohort that prenatal arsenic exposure increased expression of genes involved in various biological networks such as apoptosis, stress responses and inflammation [22].

In addition to DNA base damage, iAs can induce DNA strand breaks even at low concentrations [23]. Arsenic-induced DNA strand breaks are caused either directly by ROS attacks on the DNA bases or indirectly during the course of base excision repair [24]. Moreover, arsenic is a known inducer of chromosome aberrations [25]. Several studies have performed cytogenetic monitoring by using chromosome aberrations, sister chromatid exchanges and micronucleus (MN) assays in order to detect genotoxic effects in various populations exposed to arsenic [26]. An increase in MN frequency in arsenic-exposed individuals in peripheral blood lymphocytes in Chile, in buccal and urothelial cells in India and in buccal cells in Argentina, where the mean arsenic concentrations in drinking water were > 750 μg/L, 214.7 μg/L, and 200 μg/L, in Chile, India, and Argentina, respectively, has been reported [27].

This study investigates the consequences of in utero arsenic exposure, particularly on various types of genetic damage in newborns, from the arsenic-contaminated areas in Vietnam where the mean levels of arsenic in household drinking water varied from < 1 to 65.7 μg/L [5]. The extent of genetic damage was measured by various biomarkers of early genotoxic effects including oxidative/nitrative DNA damage (8-OHdG and 8-nitroguanine), DNA strand breaks, and micronuclei in newborns’ cord blood.

## Methods

### Study locations and subject recruitment

This study was conducted in Hanam Province, Vietnam, where the high levels of arsenic contamination in groundwater and in household drinking water levels exceed the WHO recommendation level in various locations. The selected study locations consisted of six communes of the Kim Bang district in Hanam province including Hoang Tay, Nhat Tan, Van Xa, Kha Phong, Thi Son and Ba Sao. Among these locations, drinking water in Hoang Tay and Nhat Tan villages had mean water arsenic concentrations of 65.7 and 61.7 μg/L, respectively, that exceeded both the WHO recommended level for drinking water at 10 μg/L and the Vietnamese Standards for safe drinking water of 50 μg/L. A total of 205 pregnant women were recruited during 2010–2012. Interviews regarding residential history, health problems, birth and pregnancy information (parity, abortions and complications), use of household drinking water as well as water and food consumption habits, were conducted prior to recruitment. All recruited subjects were healthy, pregnant volunteers, aged 20–40 years who had lived in the selected study locations for at least 1 year. The enrollment was at the gestation age (mean ± SD) at 25.30 ± 0.61 weeks. All subjects were undergoing natural baby delivery without birth stimulation or anesthesia to avoid the interference on DNA damage that might occur in newborns. All babies were delivered by 2013.

This study was conducted according to the recommendations of the Declaration of Helsinki [28] for international health research. Study protocols were approved by local ethic committees, and informed consent was obtained from all participating subjects before sample collection.

### Biological sample collection

Toenails and urine samples were collected from the recruited pregnant women during pregnancy at the gestational age (mean ± SD) of 25.30 ± 0.61 weeks. Umbilical cord blood samples were collected immediately after birth, and the mean gestational age (mean ± SD) at time of collection was 39.48 ± 0.12 weeks. All cord blood samples were locally processed by dividing to several fractions; 2 mL cord blood without heparin were used for collecting serum and stored at − 80 °C. The rest of cord blood samples were collected in heparinized tubes. Aliquots of these samples were stored at − 80 °C. In addition, a fraction of 2 mL of heparinized blood was immediately stabilized with 10% DMSO, placed in a cryopreservation vessel and stored at − 80 °C freezer overnight. The samples were then transferred from the cryopreservation vessel and stored at − 80 °C until analysis.

### Assessment of arsenic exposure in utero

Arsenic exposure was assessed in the mothers and their newborns. The concentrations of arsenic in the mothers’ toenails and urine samples during pregnancy and in cord blood at delivery represent maternal and newborn exposure, respectively.

#### Analysis of arsenic in nails and cord blood

Toenails were clipped from pregnant women and retained in zip-lock bags at room temperature until analysis. Briefly, all nail samples were washed by sonication with acetone and 1% (*v*/v) Triton X-100 for 10 min to remove external contamination, rinsed 5 times with deionized (DI) water, and left to dry in a clean cabinet. Samples were digested in Teflon vessels using a microwave oven (Milestone ETHOS) and analyzed for total arsenic concentrations by inductively coupled plasma mass spectrometry (ICP-MS). For quality control, a certified reference material [NCS ZC 81002b human hair; China National Analysis Center for Iron and Steel (NCS), China, with a certified value of 0.20 ± 0.02 μg/g] was analyzed. The limit of detection was 0.01 μg/g.

Cord blood samples were analyzed for arsenic concentrations using a Perkin-Elmer NexION 350S with Elemental Scientific autosampler 4DX. Our ICP-MS-DRC method was modified from previous studies [29]. Whole blood samples were thoroughly mixed, diluted 100 times with diluent (1% HNO_3_, 0.02% Triton X-100 and 1% Methanol), centrifuged for 10 min at 3500 rpm, and the supernatant reserved for analysis. A standard solution chosen to cover the expected range of arsenic concentrations in the blood samples (0.1, 0.5, and 2.5 μg/L) was used for instrument calibration. Matrix-induced interferences were corrected through the addition of selecting rhodium (Rh) 10 ng Rh per tube. Polyatomic interferences were suppressed with the instrument’s Dynamic Reaction Cell (DRC) technology feature, utilizing oxygen as a second gas. Quality control blood samples from the Institut de Sante Publique du Quebec with three different concentrations were run daily after initial calibration, after a set of 14 study samples, and again after each recalibration. Coefficients of variation for intra- and inter-precision for QC samples were 5.6 and 3.4%, respectively. All samples were analyzed in duplicate and the coefficients of variation for intra- and inter-precision were 3.6 and 14.3%, respectively. The limit of detection (LOD) of arsenic in blood samples is 0.1 μg/L.

#### Analysis of arsenic concentration in the urine

Urine samples were collected in HNO_3_-treated light-protected polyethylene tubes and stored at − 20 °C until analysis. Total arsenic concentration in urine was measured by preparing 10-fold dilutions of urine in 1% HNO_3_ (suprapure grade; Merck) and subjecting the dilutions to ICP-MS analysis, as previously described [30]. For quality control, control material (Seronorm™ Trace Elements Urine Blank, Ref 201,305, Lot OK4636, Norway with a certified value of 85–90 μg/L) was analyzed. The average concentration was 86.19 ± 2.31 μg/L (92.27% accuracy with 2.68% CV). The limit of detection was 0.04 μg/L.

For urinary arsenic speciation, urine samples, preserved with diethyldithiocarbamic acid, were diluted 10-fold with DI water and filtered through a 0.45 μm syringe filter. The filtered sample was analyzed using high performance liquid chromatography (HPLC)/ICP-MS, [Agilent Model 1100 HPLC, Gemini C18 column (150 × 4.6 mm, 5 μm), Phenomenex]. The mobile phase consists of 10 mM ammonium phosphate (Merck), 5 mM tetrabutylammonium hydroxide (Merck), and 4% methanol (HPLC grade, Merck), pH 9.2. Arsenic metabolites, including iAs (As^3+^ + As^5+^), monomethylated arsenic (MMA), dimethylated arsenic (DMA) and arsenobetaine (AB), were determined. For quality control, a SRM (SRM® 2669 level I; NIST, USA) was analyzed. The average obtained of SRM® 2669 for all 5 species showed good recovery ranging between 83.09 and 96.71% with 2.64–7.41% CV. Total urinary arsenic concentration was calculated by summing the concentrations of iAs, MMA and DMA; arsenobetaine was not included in total arsenic concentration. The LODs for iAs, MMA and DMA were 0.15, 0.13 and 0.10 μg/L, respectively. Arsenic levels in urine were adjusted with and without creatine. Urinary creatinine was measured using the Jaffe reaction with a commercial kit (Human GmbH-65,205, REF 10051, Wiesbaden, Germany). Concentrations of total arsenic and arsenic metabolites in urine were normalized to creatinine concentrations.

### Assessment of genetic damage in newborns

#### Analysis of 8-OHdG and 8-nitroguanine in serum

Levels of 8-OHdG in cord blood serum were determined using a highly sensitive 8-OHdG competitive enzyme-linked immunosorbent assay (ELISA) kit (JaICA, Japan) according to the manufacturer’s instructions. Briefly, a serum sample (300 μL) was filtered through Microcon-10 kDa Centrifugal Filter with Ultracel-10 membrane to remove high molecular weight protein, then the filtrate (50 μL) was incubated with primary monoclonal antibody (50 μL) at 4 °C overnight. Subsequently, the samples were sequentially incubated with HRP-conjugated secondary antibody (100 μL) for 1 h and chromatic solution (100 μL) for 15 min. Finally, the absorbance was measured at 450 nm. The results were expressed as ng/mL. For analysis of 8-nitoguanine, the levels of serum 8-nitroguanine were determined using a competitive ELISA kit (OxiSelect™ Nitrosative DNA/RNA Damage ELISA kit; Cell Biolabs, USA) according to the manufacturer’s instructions. Briefly, serum (50 μL) was added to the 8-nitroguanine conjugated coated microtiter plate and incubated at room temperature for 10 min. Subsequently, the sample was incubated with primary monoclonal antibody (50 μL) for 1 h and HRP-conjugated secondary antibody (100 μL) for 1 h. Then, substrate solution (100 μL) was added and the absorbance was measured at 450 nm. The results were expressed as ng/mL serum.

#### Analysis of DNA strand breaks in cord blood

For the comet assay method in frozen blood [31], 10 μL of frozen cord blood samples were quick thawed in a water bath at 37 °C and processed immediately for DNA single strand breaks. The alkaline comet assay was performed as previously described with minor modifications [32]. A total of 50 cells from each of the duplicate slides were examined randomly under an epi-fluorescence microscope (Axio Imager Z2, Zeiss, Germany). The extent of DNA damage was measured quantitatively using CometScan image analysis software (MetaSystems) and expressed as Tail length, Olive tail moment and %DNA in tail.

#### Cytokinesis block micronucleus (CBMN) assay in cord blood

Frozen cord blood (1 mL) was quick thawed in a water bath at 37 °C, then the sample was transferred into a clean tube containing 10 mL RPMI 1640 cell culture medium, mixed and centrifuged for 10 min. The pellet was resuspended in 6 mL cell culture medium (RPMI 1640 containing 20% FBS, 1% L-glutamine and 1% Pen/Strep) and incubated at 37 °C in a humidified incubator with 5% CO_2_ for 24 h. Subsequently, the cord blood cultures were subjected to a CBMN assay according to the protocol previously described [33, 34]. After 44 h of cell stimulation by phytohemagglutinin (PHA) (Murex, Dartford, UK), cytochalasin B (Sigma, USA) was added to the blood culture (final concentration of 6 μg/mL) to arrest cytokinesis. At 72 h of PHA-stimulation, the cultures were harvested, fixed and stained. To determine the frequency of MN, slides were stained with DAPI (Prolong® Gold Antifade reagent with DAPI, Cell signaling Technology, USA) and viewed under fluorescence microscope (Axio Imager Z2) equipped with Metafer MNScore software (Metasystems). A total number of 1000 mononucleated and binucleated lymphocytes were scored. The nuclear division index (NDI) was determined by staining the slides with solution containing DAPI and PI (Invitrogen, USA) at 0.25 μg/mL Vectashield Antifade Mounting Medium (Vector Laboratories, USA) and randomly scored for 500 cells. The NDI values were calculated as (M_1_ + 2 M_2_ + 3 M_3_ + 4 M_4_)/N where M_1_-M_4_ represent the number of cells with 1–4 nuclei and N is the total number of cells scored.

Our preliminary study showed that the levels of MN frequency in both mononucleated and binucleated cells and NDI in frozen blood were not different from those of fresh blood (data not shown).

### Statistical analysis

Statistcal analyses were performed using the Stata software package (version 10, StataCorp LP, College Station, TX, USA). Concentrations of arsenic that were below their corresponding LODs were imputed with the value of the LOD divided by the square root 2. There were 9 and 6 urine samples with values below the LOD for iAs and MMA, respectively. Data are expressed as mean ± SE. One-way ANOVA and Mann-Whitney U test were used to determine statistically significant differences of the study parameters in various exposure groups and between two groups, respectively. Univariate regression model was used to assess the association among the study parameters. A multivariate adjusted regression model was also used to assess the relationships between exposure variables and genetic damages in newborns. The covariates for the multivariate model were selected based on their known biological plausibility as confounders of genetic damage. The potential confounders including age (continuous), BMI (continuous), education (elementary school, secondary school, diploma and college), occupations (housewife, agricultural worker, factory worker, employee and vendor) and gestation age (continuous) at time of sample collection during pregnancy (maternal toenail and urine) and baby delivery (cord blood sample) were adjusted in the model. Arsenic concentrations were assessed for normality using the Skewness/Kurtosis test. Concentrations of arsenic in urine, toenail and cord blood were right skewed and therefore log-transformed arsenic concentration was carried out to obtain a normal distribution for use in the regression models. The associations with multivariate regression models, using residential areas and arsenic exposure (maternal toenail arsenic, urinary arsenic metabolites, cord blood arsenic) as continuous exposure variables and each of the genetic damage markers as continuous outcomes, were determined. MN frequency was z-scored prior to performing multivariate regression model. All regression coefficients were reported as standardized (z-transformed) β coefficients with 95% confidence interval (CI). In addition, a multiple testing correction was performed for multiple dependent and independent variables by False Discovery Rate (FDR) correction with the Benjamini-Hochberg procedure [35]. A *p*-value of < 0.05 was considered as a statistically significant difference for all tests.

## Results

### Demographic characteristics

Demographic characteristics of mother and infant birth outcomes are shown in Table 1. Mothers had a pregnancy BMI of 21.7 kg/m^2^ and a mean age of 26.6 years old. From the 205 pregnant women recruited, maternal arsenic exposure was stratified as low, medium and high exposure groups according to toenail arsenic levels < 0.5 μg/g, 0.5–1 μg/g and > 1 μg/g, respectively. A level of 0.5 μg/g arsenic in the toenail corresponds to regular consumption of water at the recommended level of 10 μg/L [22]. Based on maternal toenail arsenic concentrations, arsenic exposure was significantly associated with residential areas (*p* < 0.001). The majority of mothers in the low-exposed group lived in areas where arsenic contamination in drinking water was less than the WHO guideline at < 10 μg/L (22% in Ba Sao, 1.2% in Kha Phong and 58.5% in Thi Son villages). In contrast, most of the arsenic-exposed mothers in the high exposure group lived in Hoang Tay (19.7%) and Nhant Tan (60.6%) where the mean levels of arsenic in drinking water were 65.7 μg/L and 61.7 μg/L, respectively. A greater percentage of higher education level was observed in the low-exposed group, compared to medium- and high-exposed groups. Most of the women were agricultural workers (44.4%), had no complications during pregnancy (92.2%) and had no history of miscarriage (85.4%). Exposure to tobacco smoke in pregnant women was assessed by measurement of cotinine in urine; the median levels in all study groups were in the non-detectable range.

**Table 1:** Demographics characteristic of mothers and infant birth outcomes in Vietnamese pregnancy cohort

Variables	All	Maternal arsenic exposure by toenail As			
		Low (< 0.5 μg/g)	Medium (0.5–1 μg/g)	High (> 1 μg/g)	*p*-value^a^
	(*n* = 205)	(*n* = 82)	(*n* = 57)	(*n* = 66)	
Pregnancy BMI (kg/m^2^) (mean ± SD)	21.7 ± 2.2	21.5 ± 2.2	21.5 ± 2.3	22.2 ± 2.1	0.064
Maternal age (years) (mean ± SD)	26.6 ± 4.1	27.1 ± 4.3	25.7 ± 3.4	26.8 ± 4.3	0.184
Residential area (As in drinking water) [n (%)]^b^					**0.000**
Ba Sao (0.64 μg/L)	31 (15.1)	18 (22.0)	10 (17.5)	3 (4.5)	
Kha Phong (0.60 μg/L)	3 (1.5)	1 (1.2)	0	2 (3.0)	
Thi Son (1.64 μg/L)	66 (32.2)	48 (58.5)	10 (17.5)	8 (12.1)	
Hoang Tay (65.7 μg/L)	30 (14.6)	6 (7.3)	11 (19.3)	13 (19.7)	
Nhant Tan (61.7 μg/L)	67 (32.7)	4 (4.9)	23 (40.4)	40 (60.6)	
Van Xa (27.2 μg/L)	8 (3.9)	5 (6.1)	3 (5.3)	0	
Education level [n (%)]					**0.019**
Elementary school	15 (7.3)	2 (2.4)	3 (5.3)	10 (15.2)	
Secondary school	135 (65.9)	51 (62.2)	44 (77.2)	40 (60.6)	
Diploma	40 (19.5)	20 (24.4)	7 (12.3)	13 (19.7)	
College graduate	15 (7.3)	9 (11.0)	3 (5.3)	3 (4.5)	
Maternal occupation [n (%)]					0.074
Housewife	32 (15.6)	10 (12.2)	9 (15.8)	13 (19.7)	
Agricultural worker	91 (44.4)	31 (37.8)	26 (45.6)	34 (51.5)	
Factory worker	44 (21.5)	18 (22.0)	11 (19.3)	15 (22.7)	
Employee	30 (14.6)	18 (22.0)	10 (17.5)	2 (3.0)	
Vendor	8 (3.9)	5 (6.1)	1 (1.8)	2 (3.0)	
Parity [n (%)]					0.477
1 person	75 (36.6)	32 (39.0)	21 (36.8)	22 (33.3)	
2 persons	100 (48.8)	41 (50.0)	30 (52.6)	29 (43.9)	
3 persons	25 (12.2)	8 (9.8)	5 (8.8)	12 (18.2)	
≥ 4 persons	5 (2.4)	1 (1.2)	1 (1.8)	3 (4.5)	
Antenatal care services [n (%)]					0.464
Province clinic hospital	20 (9.8)	9 (11.0)	7 (12.3)	4 (6.1)	
District clinic hospital	23 (11.2)	11 (13.4)	8 (14.0)	4 (6.1)	
Health center of commune	21 (10.2)	9 (11.0)	7 (12.3)	5 (7.6)	
Private medical center	125 (61.0)	48 (58.5)	32 (56.1)	45 (68.2)	
Not having antenatal care	16 (7.8)	5 (6.1)	3 (5.3)	8 (12.1)	
Complications during pregnancy [n (%)]					0.683
No	189 (92.2)	74 (90.2)	53 (93.0)	62 (93.9)	
Yes	16 (7.8)	8 (9.8)	4 (7.0)	4 (6.0)	
History of miscarriage [n (%)]					0.409
Never	175 (85.4)	71 (86.6)	52 (91.2)	52 (78.8)	
1 time	23 (11.2)	7 (8.5)	5 (8.8)	11 (16.7)	
2 times	4 (2.0)	2 (2.4)	0	2 (3.0)	
≥ 3 times	3 (1.5)	2 (2.4)	0	1 (1.5)	
Urinary Cotinine (μg/mmol creatinine) Median (range)	nd (nd-8.03)	nd	nd (nd-8.03)	nd	
Infant birth outcomes					
Gender					0.220
Male [n (%)]	109 (53.7)	45 (54.9)	30 (53.6)	34 (52.3)	
Female [n (%)]	94 (46.3)	37 (45.1)	26 (46.4)	31 (47.7)	
Birth length (cm) (mean ± SD)	49.8 ± 2.2	50.4 ± 2.0	49.1 ± 2.8	49.8 ± 1.4	**0.001**
Birth weight (kg) (mean ± SD)	3.1 ± 0.8	3.2 ± 0.8	3.0 ± 0.8	3.0 ± 0.8	0.154
Head circumference (cm) (mean ± SD)	32.2 ± 2.7	31.9 ± 2.1	32.6 ± 4.0	32.2 ± 1.8	0.336

^a^Statistically significant difference among groups at p < 0.05 by One-way ANOVA are highlighted in bold

^b^Mean arsenic concentrations in drinking water obtained from Do et al., 2013 [5]

Infant birth outcomes stratified by maternal exposure showed that mean of birth length (49.8 cm) was significantly different among groups (*p* < 0.001). The mean birth length of the low-exposed group (50.40 cm) was significantly higher than that of the medium- (49.1 cm, *p* < 0.001) and the high-exposed groups (49.8 cm, *p* < 0.01). In contrast, birth weight, head circumference and gender of newborns were not associated with maternal arsenic exposure.

### Assessment of arsenic exposure in mother and newborns

Maternal arsenic exposure level was determined by toenail arsenic concentration as a biomarker of long term exposure, and urinary arsenic concentration as a biomarker of recent exposure (Table 2). Maternal toenail arsenic concentrations were significantly different among study groups (*p* < 0.001). The mean levels of arsenic in toenails in pregnant subjects in the medium-exposed group (0.73 μg/g) and high-exposed group (1.92 μg/g) were significantly higher than that of the low-exposed group (0.30 μg/g, *p* < 0.001). The mean urinary arsenic concentrations in mothers, measured as the sum of iAs and its metabolites (i.e. MMA and DMA) increased with increasing exposure levels. When compared to the low-exposed group, pregnant women in the high-exposed group had a significant increase in concentrations of total arsenic in urine by 64% (*p* < 0.01) and urinary metabolites of MMA and DMA by 68% (*p* < 0.001) and 43% (*p* < 0.01), respectively.

**Table 2:** Arsenic exposure in mothers and newborns

Parameters	Maternal arsenic exposure by toenail As			*p*-value ^a^
	Low (< 0.5 mg/g)	Medium (0.5–1 mg/g)	High (> 1 mg/g)	
	(*n* = 82)	(*n* = 57)	(*n* = 66)	
Maternal toenail As (μg/g)	0.30 ± 0.01^b^	0.73 ± 0.02^***^	1.92 ± 0.13^***, ###^	**0.000**
	0.29 (0.10–0.49)^c^	0.71 (0.50–0.99)	1.55 (1.00–6.07)	
Maternal urinary As				
Total arsenic (μg/L)	28.20 ± 2.27	40.05 ± 3.69^*^	60.71 ± 6.23^***,##^	**0.000**
	25.26 (2.16–130.23)	29.26 (8.48–111.05)	50.55 (10.03–328.90)	
Total arsenic (μg/g creatinine)	57.46 ± 4.25	67.95 ± 4.58	93.91 ± 7.42^***, ##^	**0.013**
	46.68 (1.57–162.39)	61.88 (13.39–166.66)	75.59 (16.57–339.59)	
Urinary As metabolites				
iAs (μg/L)	3.48 ± 0.34	3.38 ± 0.32	4.71 ± 0.64^**, #^	**0.014**
	2.96(nd-17.13)	2.96(nd-9.67)	3.05 (0.68–38.26)	
MMA (μg/L)	4.60 ± 0.43	5.57 ± 0.51^*^	7.74 ± 0.81^***^	**0.000**
	3.98(nd-18.42)	4.30(nd-16.82)	6.03 (0.13–40.13)	
DMA (μg/L)	32.90 ± 2.84	34.69 ± 3.34	47.14 ± 4.90^**^	**0.010**
	25.26(1.59–140.92)	26.28(9.08–115.14)	36.17 (5.04–247.81)	
Umbilical cord blood As (μg/L)	1.70 ± 0.09	2.09 ± 0.13	2.46 ± 0.21^**^	**0.000**
	1.50 (0.17–4.60)	1.78 (0.85–6.22)	2.05 (0.89–9.72)	

^a^Statistically significant difference among groups at *p* < 0.05 by One-way ANOVA are highlighted in bold

Values are expressed as mean ± SE (^b^) and median (minimum - maximum) (^c^)

^*, **, ***^ Statistically significant difference from corresponding low-exposed group at *p* < 0.05, < 0.01 and < 0.001, respectively by Mann-Whitney U test

^#, ##, ###^Statistically significant difference from corresponding medium-exposed group at *p* < 0.05, < 0.01 and < 0.001, respectively by Mann-Whitney U test

**Fig. 1:**
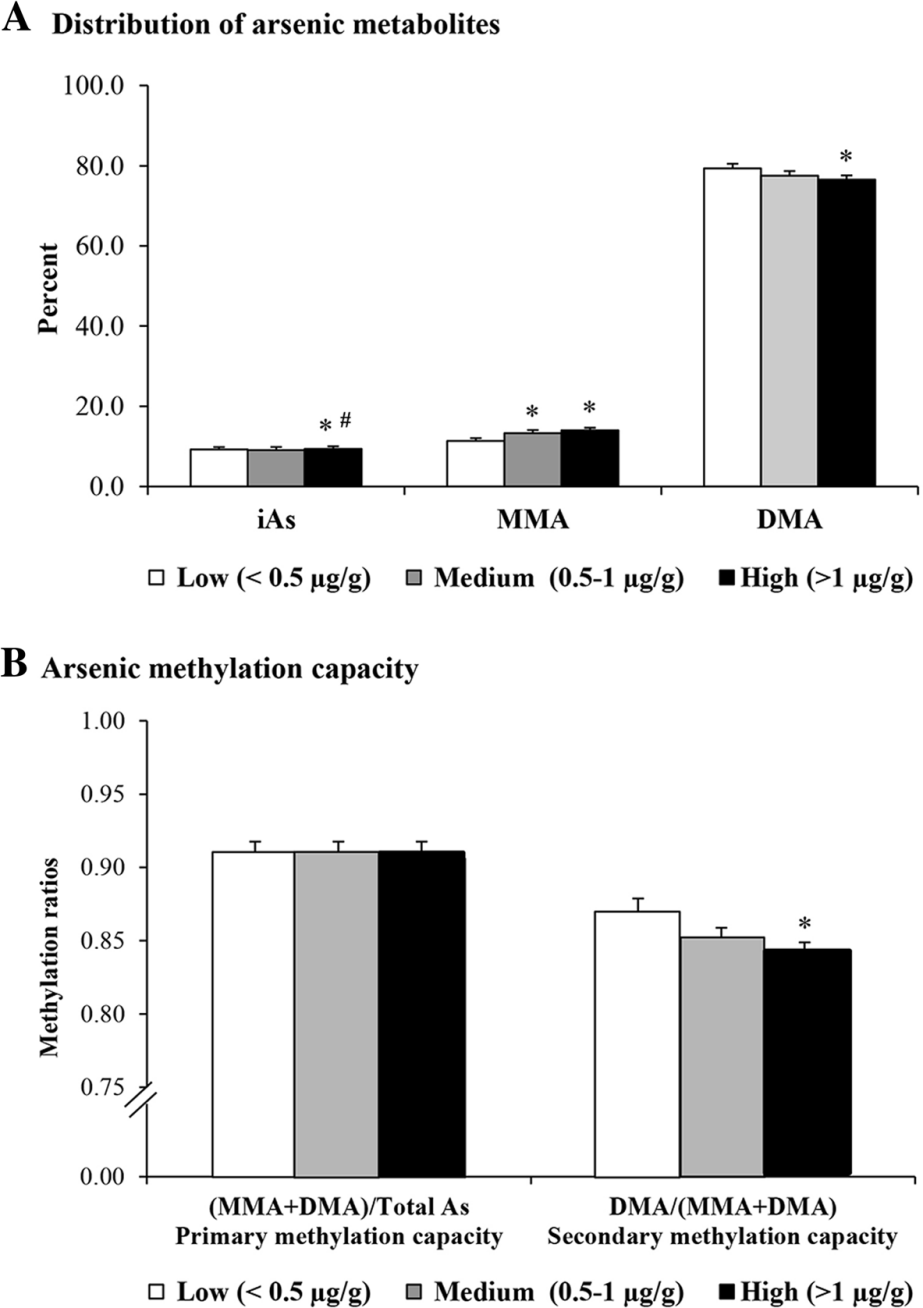
Distribution of arsenic metabolites in maternal urinary samples (**a**) and Methylation capacity of arsenic in urine (**b**) Each bar represents mean ± SE.*^,^ represents significant difference from low arsenic-exposed group at *p* < 0.05. #^,^ represents significant difference from medium arsenic-exposed group at *p* < 0.05

As shown in Fig. 1a, the distribution of iAs and its metabolites in urine in all study groups is in the order of DMA > MMA > iAs. The percentage of distribution of iAs and MMA in the high-exposed group was significantly increased, but DMA decreased, when compared to the corresponding arsenic metabolites in the low-exposed group.

To compare arsenic methylation among groups, methylation capacity ratios were determined. Methylation indices, primary methylation (total methylated metabolites (MMA + DMA)/total arsenic) and secondary metabolites (DMA/total metabolites), were measured to assess arsenic methylation capacity. In the high arsenic-exposed groups, the mean level of urinary secondary methylation was significantly decreased compared to those of the low- and the medium-exposed groups (*p* < 0.05) (Fig. 1b). The results indicated that the ability to methylate arsenic metabolites was lower with higher arsenic exposure.

Arsenic exposure in newborns, determined by cord blood arsenic concentration, increased significantly with increasing levels of maternal arsenic exposure (*p* < 0.001) (Table 2). The mean cord blood arsenic concentration from the high arsenic-exposed mothers (2.46 μg/L) was significantly higher than those from the low-exposed group by 44% (1.70 μg/L; *p* < 0.001) and the medium-exposed group by 17% (2.09 μg/L). An increase in cord blood arsenic concentration in relation to maternal arsenic exposure levels confirmed that arsenic exposure occurs in newborns in utero as a result of maternal exposure during pregnancy.

### DNA damage in arsenic-exposed newborns

To assess the impacts of arsenic exposure in utero on early genotoxic effects in newborns in a dose-dependent manner, levels of DNA damage including 8-OHdG, 8-nitroguanine and DNA strand breaks were determined in relation to maternal arsenic exposure levels. Table 3 shows various types of DNA damage in newborns, all of which significantly increased with increasing levels of maternal arsenic exposure. Levels of 8-OHdG in cord blood from the high arsenic-exposed group was higher than those from the medium and the low arsenic-exposed groups (*p* < 0.01). Consistent with 8-OHdG findings, the mean level of 8-nitroguanine increased by 16% in the medium-exposed group (183.21 ng/mL) and by 45% in the high arsenic-exposed group (229.94 ng/mL; *p* < 0.05), when compared to that from the low-arsenic exposed group (157.66 ng/mL). DNA strand breaks in cord blood also increased with increasing maternal exposure levels. Levels of DNA strand break measured as tail length, Olive tail moment and %DNA in tails significantly increased in the medium- and the high arsenic-exposed groups, when compared to the low-exposed group.

**Table 3:** DNA damage in cord blood of newborns exposed to arsenic in utero

Types of DNA damage	Maternal arsenic exposure by toenail As			FDR- adjusted *p*-value^a^
	Low (< 0.5 μg/g)	Medium (0.5–1 μg/g)	High (> 1 μg/g)	
8-OHdG (ng/mL)	0.24 ± 0.01^b^	0.26 ± 0.01	0.28 ± 0.01^**^	**0.010**
	0.24 (0.13–0.44)^c^	0.26 (0.18–0.49)	0.26 (0.19–0.55)	
	(*n* = 81)	(*n* = 52)	(*n* = 61)	
8-Nitroguanine (ng/mL)	157.66 ± 13.85	183.21 ± 17.90	229.94 ± 23.34^*^	**0.032**
	117.20 (13.59–426.51)	154.99 (22.51–540.60)	178.77 (26.73–831.84)	
	(*n* = 78)	(*n* = 53)	(*n* = 58)	
DNA strand breaks				
Tail length (μm)	2.37 ± 0.08	2.81 ± 0.10^**^	3.04 ± 0.09^***^	**0.000**
	2.38 (0.65–4.11)	2.74 (1.13–4.03)	2.98 (1.69–4.92)	
	(*n* = 73)	(*n* = 48)	(*n* = 54)	
Olive tail moment (μm)^d^	0.20 ± 0.01	0.24 ± 0.01^**^	0.27 ± 0.01^***^	**0.000**
	0.19 (0.07–0.39)	0.22 (0.07–0.44)	0.26 (0.11–0.49)	
	(n = 73)	(n = 48)	(n = 54)	
%DNA in tail	1.36 ± 0.06	1.62 ± 0.08^*^	1.94 ± 0.10^***, ##^	**0.000**
	1.28 (0.53–3.00)	1.51 (0.37–2.88)	1.82 (0.48–4.05)	
	(*n* = 73)	(*n* = 48)	(*n* = 54)	

^a^The raw *p*-values were subjected to multiple testing correction using FDR-adjusted *p*-value; Statistically significant difference among groups at *p* < 0.05 by One-way ANOVA are highlighted in bold

Values are expressed as mean ± SE (b) and Median (minimum - maximum) (c)

^*, **, ***^ Statistically significant difference from corresponding low- exposed group at *p* < 0.05, < 0.01 and < 0.001, respectively by Mann-Whitney U test

^##^Statistically significant difference from corresponding medium-exposed group at *p* < 0.01 by Mann-Whitney U test

^d^Olive tail moment is the product of the tail length and the fraction of total DNA in the tail

### Cytogenetic damage in arsenic-exposed newborns

The cytogenetic effect of arsenic exposure in utero was determined by MN assay in cord blood. As shown in Table 4, MN frequency, analyzed by cytokinesis block micronucleus (CBMN), was measured as MN in mononucleated cells and in binucleated cells as well as nuclear division index (NDI). MN frequency in mononucleated cells was 0.16, 0.48 and 0.60 in low-, medium- and high-exposed groups, respectively. MN frequency in mononucleated lymphocytes increased with increasing maternal arsenic exposure levels. The MN frequency in the medium- and the high arsenic-exposed groups was significantly higher than that of low-exposed group at *p* < 0.01 and *p* < 0.001, respectively.

**Table 4:** Micronucleus frequency in cord blood of newborns exposed to arsenic in utero

Parameters	Maternal arsenic exposure by toenail As			FDR- adjusted *p*-value^a^
	Low (< 0.5 μg/g)	Medium (0.5–1 μg/g)	High (> 1 μg/g)	
MN in mononucleated cells (per 1000 cells)	0.16 ± 0.04^b^	0.48 ± 0.08^**^	0.60 ± 0.09^***^	**0.000**
	0.00 (0.00–1.00)^c^	0.00 (0.00–2.00)	1.00 (0.00–2.00)	
	(*n* = 70)	(*n* = 46)	(*n* = 52)	
MN in binucleated cells (per 1000 cells)	1.96 ± 0.21	2.26 ± 0.34	3.08 ± 0.41^*^	**0.037**
	2.00 (0.00–12.0)	2.00 (0.00–13.0)	2.00 (0.00–15.0)	
	(*n* = 70)	(*n* = 46)	(*n* = 52)	
Nuclear division index (NDI)	1.94 ± 0.01	1.97 ± 0.02	1.94 ± 0.01	0.117
	1.92 (1.75–2.19)	1.97 (1.79–2.75)	1.95 (1.59–2.07)	
	(*n* = 70)	(*n* = 46)	(*n* = 52)	

^a^The raw *p*-values were subjected to multiple testing correction using FDR-adjusted *p*-value; Statistically significant difference among groups at p < 0.05 by One-way ANOVA are highlighted in bold

Values are expressed as mean ± SE (b) and Median (minimum - maximum) (c)

^*, **, ***^ Statistically significant difference from corresponding low- exposed group at *p* < 0.05, < 0.01 and < 0.001, respectively by Mann-Whitney U test

The frequency of MN in binucleated cells also increased with increasing exposure levels. A significant increase in MN frequency was observed in high-arsenic exposed groups, compared to low-exposed group (3.08 vs 1.96, *p* < 0.05). However, NDI value, a marker of cell proliferation which is a measure of general cytotoxicity, was not significantly different among groups.

### Associations between arsenic exposure and genetic damage

Univariate analysis was performed to evaluate the associations among study parameters. As shown in Table 5, maternal arsenic exposure measured as arsenic concentration in toenails was significantly associated with all types of genetic damage in newborns, including DNA base damage (8-OHdG; β = 0.068; 95% CI: 0.023, 0.133, *p* < 0.01, 8-nitroguanine; β =0.244; 95% CI: 0.078, 0.410, *p* < 0.01) and DNA strand breaks (Tail length; β = 0.112; 95% CI: 0.056 0.168, *p* < 0.001, Olive tail moment; β = 0.083; 95% CI: 0.017, 0.149, *p* < 0.05, % DNA in tail; β = 0.131; 95% CI: 0.056, 0.207, *p* < 0.01), as well as MN frequency (mononucleated cells; β = 0.698; 95% CI: 0.272, 1.124, *p* < 0.01). Levels of maternal urinary arsenic and arsenic metabolites were also significantly associated with DNA strand breaks and MN frequency. Arsenic concentrations in cord blood had a significant association with all parameters of DNA strand breaks (Tail length; β = 0.192; 95% CI: 0.094, 0.290, *p* < 0.001, Olive tail moment; β = 0.256; 95% CI: 0.140, 0.373, *p* < 0.001, % DNA in tail; β = 0.244; 95% CI: 0.111, 0.377, *p* < 0.001) and MN frequency (binucleated cells; β = 0.774; 95% CI: 0.560, 0.988, *p* < 0.001).

**Table 5:** Univarite analysis of associations among the study parameters

	Coefficient β (95% CI)									
	DNA base damage		DNA strand breaks			Micronucleus frequency		Arsenic concentration		
	8-OHdG	8-nitro	Tail length	Olive tail moment	% DNA in tail	Mono	Bi	Urine	Cord blood	Toenail
		guanine				nucleated	nucleated	(μg/g creatinine)		
Arsenic exposure										
Maternal toenail	0.068^**^	0.244^**^	0.112^***^	0.083^*^	0.131^**^	0.698^**^	− 0.045	0.225^***^	0.126^**^	
	(0.023,0.133)	(0.078,0.410)	(0.056,0.168)	(0.017,0.149)	(0.056,0.207)	(0.272,1.124)	(−0.167,0.077)	(0.113,0.336)	(0.043,0.209)	
Maternal urine										
Total As	0.003	− 0.017	0.058	0.135^**^	0.100^*^	0.378	0.128			
	(−0.054,0.061)	(− 0.229,0.196)	(− 0.014,0.129)	(0.051,0.220)	(0.004,0.197)	(− 0.166,0.922)	(− 0.028,0.084)			
iAs	−0.021 (−0.079,0.037)	0.155 (−0.060,0.371)	−0.009 (− 0.081,0.064)	0.043 (− 0.043,0.129)	0.02 (− 0.078,0.118)	−0.085 (− 0.637,0.468)	0.051 (− 0.107,0.209)	−0.054 (− 0.205,0.097)	0.007 (− 0.103,0.117)	−0.184 (− 0.375,0.007)
MMA	0.019 (−0.039,0.077)	0.141 (−0.075,0.356)	0.010 (− 0.063,0.082)	0.047 (− 0.039,0.133)	0.039 (− 0.059,0.137)	0.496 (− 0.056,1.048)	0.115 (− 0.043,0.273)	−0.107 (− 0.257,0.043)	0.054 (− 0.056,0.163)	0.215^*^ (0.025,0.405)
DMA	− 0.01 (− 0.075,0.055)	− 0.238 (− 0.475,0.003)	0.011 (− 0.070,0.092)	− 0.057 (− 0.153,0.039)	− 0.025 (− 0.135,0.084)	− 0.146 (− 0.763,0.470)	− 0.070 (− 0.246,0.106)	0.445^***^ (0.290,0.600)	− 0.097 (− 0.218,0.025)	0.003 (−0.212,0.218)
Cord blood	−0.056 (−0.135,0.023)	−0.086 (−0.379,0.206)	0.192^***^ (0.094,0.290)	0.256^***^ (0.140,0.373)	0.244^***^ (0.111,0.377)	0.456 (−0.292,1.204)	0.774^***^ (0.560,0.988)	0.042 (−0.163,0.246)		
*DNA damage*										
8-OHdG		0.025 (−0.021,0.071)	0.082 (−0.083,0.247)	−0.119 (− 0.356,0.119)	0.040 (− 0.148,0.229)	0.016 (−0.007,0.038)	−0.035 (− 0.096,0.026)			
8-nitroguanine			0.043 (−0.563,0.649)	0.170 (−0.700,1.040)	0.459 (− 0.226,1.144)	−0.008 (− 0.091,0.075)	−0.063 (−0.286,0.160)			
*DNA strand breaks*										
Tail length				0.432^***^ (0.201,0.663)	0.063 (−0.128,0.255)	0.002 (−0.021,0.026)	0.064^*^ (0.003,0.125)			
Olive tail moment					0.574^***^ (0.482,0.666)	0.033^***^ (0.018,0.048)	0.008 (−0.035,0.051)			
%DNA in tail						0.003 (−0.018,0.023)	0.019 (−0.036,0.074)			

^*, **,***^ Statistically significant association at *p* < 0.05, *p* < 0.01 and *p* < 0.001, respectively

β represents standardized coefficient; 95% CI represents 95% confidence interval (CI)

Olive tail moment is the product of the tail length and the fraction of total DNA in the tail

In addition, a significant association was observed between DNA strand breaks and MN frequency. However, 8-OHdG and 8-nitroguanine were not associated with DNA strand breaks and MN frequency. Arsenic concentrations in maternal toenail concentration was also significantly associated with arsenic concentrations in maternal urinary (β = 0.225; 95% CI: 0.113, 0. 336, *p* < 0.001) and cord blood (β = 0.126; 95% CI: 0.043, 0.209, *p* < 0.01).

Multivariable-adjusted regression analysis was additionally performed to account for potential confounders. As shown in Table 6, the model adjusted for covariates of age, BMI, education, occupation and gestational age at time of sample collection during pregnancy (maternal toenail and urine) and baby delivery (cord blood sample) were analyzed in association with various types of genetic damage. After adjustment for confounders, maternal toenail arsenic was significantly associated with DNA base damage (8-OHdG; β = 0.234; 95% CI: 0.089, 0.379, *p* = 0.010 and 8-nitroguanine; β = 0.210; 95% CI: 0.064, 0.356, *p* = 0.031), DNA strand breaks (Tail length; β = 0.360; 95% CI: 0.221, 0.499, *p* = 0.000), Olive moment; β = 0.192; 95% CI: 0.045, 0.339, *p* = 0.000, %DNA in tai; β = 0.273; 95% CI: 0.124, 0.422, *p* = 0.000) and MN frequency (mononucleated cells; β = 0.325; 95% CI: 0.177, 0.472, *p* = 0.000). Maternal urinary arsenic was not associated with DNA damage; however, significant associations were observed between urinary total arsenic and DNA strand breaks (Olive tail moment, β = 0.232; 95% CI: 0.089, 0.076, *p* = 0.000). Cord blood arsenic was significantly associated with DNA strand breaks, measured as Tail length (β = 0.270; 95% CI: 0.131, 0.409, *p* = 0.001), Olive moment (β = 0.316; 95% CI: 0.178, 0.454, *p* = 0.000) and %DNA in tail (β = 0.264; 95% CI: 0.125, 0.404, *p* = 0.001), and binucleated MN frequency (β = 0.519; 95% CI: 0.380, 0.657, *p* = 0.000), but not 8-OHdG or 8-nitroguanine.

**Table 6:** Multivariate regression analyses between arsenic exposure and early genotoxic effects in newbornss

	Coefficient β [adjusted *p*-value] (95%CI)						
	DNA base damage		DNA strand breaks			Micronucleus frequency	
	8-OHdG	8-Nitroguanine	Tail Length	Olive tail moment	%DNA in tail	Mononucleated	Binucleated
Maternal Toenail (μg/L)	0.234 (0.089, 0.379) **[0.010]**	0.210 (0.064, 0.356) **[0.031]**	0.360 (0.221, 0.499) **[0.000]**	0.192 (0.045, 0.339) **[0.000]**	0.273 (0.124, 0.422) **[0.000]**	0.325 (0.177, 0.472) **[0.000]**	−0.029 (−0.175, 0.118) [0.958]
Maternal Urine Total As (μg/g creatinine)	0.003 (−0.153, 0.158) [0.974]	−0.063 (−0.219, 0.094) [0.518]	0.142 (− 0.001, 0.285) [0.140]	0.232 (0.089, 0.376) **[0.000]**	0.165 (0.020, 0.310) [0.073]	0.100 (−0.056, 0.256) [0.220]	0.090 (− 0.052, 0.232) [0.146]
iAs (μg/L)	−0.038 (−0.183, 0.107) [0.974]	0.109 (−0.037, 0.255) [0.423]	0.021 (− 0.114, 0.156) [0.781]	0.093 (− 0.042, 0.228) [0.112]	0.06 (−0.077, 0.197) [0.359]	−0.034 (− 0.213, 0.144) [0.339]	0.050 (− 0.119, 0.219) [0.146]
MMA (μg/L)	0.012 (−0.137, 0.161) [0.974]	0.076 (−0.074, 0.226) [0.518]	0.054 (− 0.085, 0.192) [0.538]	0.087 (− 0.052, 0.226) **[0.027]**	0.073 (−0.068, 0.214) [0.268]	0.163 (0.016, 0.311) [0.091]	0.153 (0.011, 0.295) [0.114]
DMA (μg/L)	−0.006 (−0.155, 0.143) [0.974]	−0.060 (−0.209, − 0.090) [0.518]	0.085 (−0.053, 0.223) [0.538]	0.003 (− 0.148, 0.153) [0.056]	0.029 (− 0.123, 0.181) [0.375]	−0.012 (−0.208, 0.183) [0.255]	−0.011 (− 0.199, 0.177) [0.227]
Cord blood (μg/L)	−0.105 (−0.254, 0.044) [0.503]	−0.026 (−0.177, 0.125) [0.732]	0.270 (0.131, 0.409) **[0.001]**	0.316 (0.178, 0.454) **[0.000]**	0.264 (0.125, 0.404) **[0.001]**	0.094 (−0.054, 0.242) [0.255]	0.519 (0.380, 0.657) **[0.000]**

Model was adjusted for the covariates including age, BMI, education, occupation, gestational age of sample collection during pregnancy (urine and nail samples) and baby delivery (cord blood sample)

β represents standardized coefficient; 95% CI represents 95% confidence interval (CI)

The raw p-values were subjected to multiple testing correction using FDR-adjusted *p*-value and the statistically significant are highlighted in bold

Olive tail moment is the product of the tail length and the fraction of total DNA in the tail

## Discussion

Our study clearly revealed the detrimental impacts of arsenic exposure during pregnancy which results in various types of genetic damage in newborns in a dose-dependent manner. If such damage persists, it may contribute to the initiation of cancer which may develop later in life.

Arsenic exposure in mothers and their newborns determined as arsenic accumulation in the nails is a sensitive biomarker of long-term arsenic exposure [1]. In this study, maternal toenail arsenic concentrations were significantly associated with residential areas (*p* < 0.001). The majority of mothers in the high arsenic-exposed group lived in the Hoang Tay and Nhat Tan villages where arsenic contamination in drinking water was > 60 μg/L [5]. In contrast, more than 80% of the mothers in the low arsenic exposed-group lived in Ba Sao, Kha Phong and Thi Son where arsenic contamination in drinking water is < 10 μg/L [5]. These results suggested that drinking water is the major source contributing to arsenic exposure in this study. Urinary arsenic concentration is a biomarker of more recent arsenic exposure because it has a half-life of only 4 days in humans [36] and the levels correlated with arsenic intake and dietary sources [37]. In this study, maternal urinary arsenic concentration expressed as μg/g creatinine was significantly associated with maternal toenail concentration (*p* < 0.001) and cord blood arsenic concentration (*p* < 0.01).

Cord blood arsenic concentrations reflect chronic arsenic exposure of the fetus during pregnancy [38]. In this study, mean cord blood arsenic concentrations were 1.70, 2.09 and 2.46 μg/L in low-, medium- and high-arsenic exposed groups, respectively. Cord blood arsenic concentration was significantly associated with that of maternal toenail arsenic (*r* = .295, *p* < 0.001) suggesting an association between fetal and maternal arsenic exposure via drinking water. Furthermore, a significant correlation of arsenic exposure in both mothers and their newborns was in agreement with other epidemiological studies that maternal blood and cord blood arsenic concentrations are highly correlated [39].

The extent of arsenic toxicity in humans has been reported to be dependent on an individual’s capacity to methylate arsenic. The profile of arsenic species reflects the methylation capacity of ingested iAs, and in turn the related toxicity in the body [40]. Methylated arsenic metabolites can be expressed as a primary methylation index (PMI; ratio of methylated metabolite concentration to total arsenic concentration) and a secondary methylation index (SMI; ratio of DMA to total methylated metabolites concentration). Our results showed that the group with high exposure had a significant reduction in arsenic methylation capacity, indicated by decreased values for SMI in urine samples compared to the lowest exposed group. Consistent with our results, prior studies have shown that the capacity to methylate MMA to DMA is reduced with increasing exposures [41] and the association between arsenic exposure and decreased methylation capacity across various age groups and doses has been reported among arsenic-exposed group in China [42]. Lower arsenic methylation capacity, characterized by higher urinary excretion of iAs and MMA as well as higher MMA% and lower SMI, has been associated with increased risk of skin lesion, hypertension, and bladder cancer [41]. A recent prospective case-control study in arsenic-exposed children revealed that arsenic methylation capacity is dose-dependently associated with developmental delays and other indicators of children’s health [43]. In addition, a reduction in methylation capacity in exposed populations has been associated with arsenic-induced ROS generation and higher susceptibility to oxidative DNA damage [44]. This association was in line with our previous study [21] in which a significant reduction in arsenic methylation capacity in children exposed to arsenic in utero and continued exposure during early childhood had a significant increase in oxidative DNA damage measured as increased 8-OHdG and decreased hOGG1 expression in salivary samples.

Cumulative evidence has shown that the fetus is extremely vulnerable to effects of chemicals when exposure occurs in utero. This exposure can affect the health of the fetus before and after birth. Various mediators involved in stress such as hormones and cytokines derived from the maternal body are generally transported into cord blood [45]. The increased 8-OHdG, 8-nitroguanine, DNA strand breaks and MN frequency in the newborns suggested that transplacental transfer of arsenic and its toxic intermediate and metabolites from the mother contribute to genetic damage in the newborns.

In recent years, 8-OHdG has been used in many studies not only as a biomarker of oxidative DNA damage but also as an indicator of risk for many diseases including cancer. 8-OHdG can be repaired by hOGG1, which cleaves damaged guanosine from DNA and thereafter it is secreted to extracellular fluids [46]. The existing data suggest that extracellular 8-OHdG levels are not affected by diet, cell death or artifact formation [47]. Serum 8-OHdG, a degraded and/or cleaved oxidative product of cellular DNA released to the serum, is associated with the prognosis of several carcinomas such as small cell lung carcinoma [48], endometrioid-type ovarian cancer [49] and acute leukemia in children [50]. In this study, levels of 8-OHdG in cord blood serum was increased in relation to maternal arsenic exposure and significantly associated with levels of arsenic in maternal toenails (*p* < 0.01).

In the current investigation, increased maternal arsenic exposure during pregnancy was also associated with increased levels of 8-nitroguanine in cord blood serum. These findings were in line with those from our recent study in a Thai cohort [20] that arsenic exposure in utero and continued exposure during childhood increased levels of urinary 8-nitroguanine in exposed newborns and in children through their early life. In addition, the levels of 8-nitroguanine was significantly correlated with promoter hypomethylation and increased expression of *COX2*, *EGR1*, and *SOC3*, all of which are involved in inflammation. These effects suggest the mechanisms through which arsenic exposure in utero and early life resulted in inflammation-induced DNA damage, which may contribute to disease and cancer development in later life.

Single strand DNA breaks are the most common lesions induced by exogenous genotoxic substances. A recent in vitro study also demonstrated that arsenite treatment in mouse thymus cells at environmentally relevant levels induces dose-dependent genotoxicity; increases DNA strand breaks by inhibiting poly (ADP-ribose) polymerase (PARP) activity, which is involved in BER for single strand breaks and oxidative DNA damage, at a low concentration (50 nM), and produces oxidative stress at higher concentrations (500 nM) [51]. The present study suggests an association between prenatal arsenic exposure and a significant increase in DNA strand breaks in newborns’ cord blood in a dose-dependent manner. Multivariate regression analysis showed that all study parameters of DNA strand breaks in umbilical cord blood were significantly associated with arsenic concentrations in cord blood and maternal toenails.

Arsenic is a known agent that causes chromosome breakage (clastogen) and affects the spindle fibers that induce chromosome loss (aneugen) which could give rise to incorrect chromosome segregation, leading to micronuclei (MN) formation [52]. Therefore, Because increased MN frequencies in T lymphocytes from adults have been shown to be predictive for cancer, therefore MN formation is another cytogenetic biomarker that has been widely used as a biomarker of early genetic effects [53] and potential biomarker of cancer risk [54]. MN in mononucleated and binucleated lymphocytes are different but complementary measures of genetic damage [34]. The presence of MN in mononucleated cells indicates chromosome breakage/loss before the blood was sampled and reflects damage accumulated during pregnancy (in utero exposure only) [34]. Higher levels of MN in binucleated cord blood lymphocytes were significantly associated with shorter telomere length (*p* = 0.039) [55]. Short telomeres have been suggested to be a potential cancer predisposition factor, indicative of increased genomic instability [43].

In this study, increased MN frequencies with respect to maternal exposure to arsenic were found in the cord blood of arsenic-exposed newborns. Elevated levels of MN frequency in mononucleated cells and binucleated cells in umbilical cord blood was significantly associated with arsenic concentrations in maternal toenails and cord blood suggesting the possible effects of maternal arsenic exposure on genomic instability in fetuses in utero. A study from the European Union (EU) Project, the NewGeneris Cohort, reported large inter-individual variations of MN frequency measured in cord blood within and between cohorts, with the highest level observed in Greece and the lowest in the United Kingdom; the mean levels of MN frequency were 1.79 and 0.55 per 1000 binucleated cells, respectively [54]. In our study, the mean levels of MN frequency were 1.96, 2.26, and 3.08 per 1000 binucleated cells in cord blood samples from low-, medium- and high-maternal arsenic exposures, respectively. These levels of MN frequency were higher than those from the EU project, even at the low maternal-arsenic exposed group. The MN frequency in this Vietnamese cohort was also significantly correlated with DNA strand breaks, but not DNA damage.

Multivariable adjusted regression analysis showed a significant association between various types of genetic damage (DNA damage, DNA strand breaks and mononucleated MN frequecy) and maternal toenail arsenic. Cord blood arsenic was significantly associated with DNA strand breaks and binucleated MN frequency. The present study suggested an association between maternal arsenic exposure and genetic damage in newborns. However, this study has some limitations, such as possible residual confounding factors that may influence genetic damage, e.g., co-exposure to other contaminants during pregnancy and maternal genotypes. Additionally, newborns’ urine samples could not be obtained to determine the efficiency of arsenic metabolism in newborns to assess the association of arsenic methylation capacity and genetic damage. Future research is needed for the follow-up study to examine the links between in utero arsenic exposure, genetic damage in newborns and disease development later in life.

## Conclusions

The results in this study provide evidence to support an association between arsenic exposure in utero and various types of genetic damage in the newborns as determined by 8-OHdG, 8-nitroguanine and DNA strand breaks as well as MN frequency. Importantly, these effects are dose-dependent. Increased DNA damage and micronuclei in the newborn may increase risk for diseases, including cancer development later in life. The use of an integrated approach of biomarkers of arsenic exposure and early genotoxic effects provides a better understanding and mechanistic insight into the health risks of in utero arsenic exposure. The information obtained here highlights the importance of prevention/intervention of arsenic exposure during pregnancy and the need for effective strategies to reduce the risk for development of diseases associated with such exposure.

## Abbreviations

8-OHdG: 8-hydroxydeoxyguanosine
BMI: Body mass index
CBMN: Cytokinesis-block micronucleus
DMA: Dimethylated arsenic
ELISA: Enzyme linked immunosorbent assay
EU: European Union
*hOGG1*: Human 8-oxoguanine DNA glycosylase 1
iAs: Inorganic arsenic
ICP-MS: Inductively coupled plasma mass spectrometry
MMA: Monomethylated arsenic
MN: Micronucleus
NDI: Nuclear division index
PMI: Primary methylation index
RNS: Reactive nitrogen species
ROS: Reactive oxygen species
SMI: Secondary methylation index
WHO: World Health Organization
