# Correction to: Exposure to arsenic in utero is associated with various types of DNA damage and micronuclei in newborns: a birth cohort study

**DOI:** 10.1186/s12940-019-0504-4

**Published:** 2019-07-24

**Authors:** Panida Navasumrit, Krittinee Chaisatra, Jeerawan Promvijit, Varabhorn Parnlob, Somchamai Waraprasit, Chalida Chompoobut, Ta Thi Binh, Doan Ngoc Hai, Nguyen Duy Bao, Nguyen Khac Hai, Kyoung-Woong Kim, Leona D. Samson, Joseph H. Graziano, Chulabhorn Mahidol, Mathuros Ruchirawat

**Affiliations:** 1 Laboratories of Environmental Toxicology/Chemical Carcinogenesis, Chulabhorn Research Institute, Laksi, Bangkok, 10210 Thailand; 2 Center of Excellence on Environmental Health and Toxicology, CHE, Ministry of Education, Ratchathewi, Bangkok, 10400 Thailand; 3 National Institute of Occupational and Environmental Health, Hanoi, Vietnam; 4 International Environmental Research Center, Gwangju Institute of Science and Technology, Gwangju, South Korea; 5 Center for Environmental Health Sciences, Massachusetts Institute of Technology, Cambridge, USA; 6 Department of Environmental Health Sciences, Columbia University, New York, USA

**Correction to: Environ Health**

**https://doi.org/10.1186/s12940-019-0481-7**

Following publication of the original article [1], the author reported that incorrect version of Tables 1, 3, 5 and 6 were published.

**Table 1:** Demographic characteristics of mothers and infant birth outcomes in Vietnamese pregnancy cohort

Variables	All	Maternal arsenic exposure by toenail As			
		Low (< 0.5 μg/g)	Medium (0.5–1 μg/g)	High (> 1 μg/g)	*p*-value^a^
	(*n* = 205)	(*n* = 82)	(*n* = 57)	(*n* = 66)	
Pregnancy BMI (kg/m^2^) (mean ± SD)	21.7 ± 2.2	21.5 ± 2.2	21.5 ± 2.3	22.2 ± 2.1	0.064
Maternal age (years) (mean ± SD)	26.6 ± 4.1	27.1 ± 4.3	25.7 ± 3.4	26.8 ± 4.3	0.184
Residential area (As in drinking water) [n (%)]^b^					**0.000**
Ba Sao (0.64 μg/L)	31 (15.1)	18 (22.0)	10 (17.5)	3 (4.5)	
Kha Phong (0.60 μg/L)	3 (1.5)	1 (1.2)	0	2 (3.0)	
Thi Son (1.64 μg/L)	66 (32.2)	48 (58.5)	10 (17.5)	8 (12.1)	
Hoang Tay (65.7 μg/L)	30 (14.6)	6 (7.3)	11 (19.3)	13 (19.7)	
Nhant Tan (61.7 μg/L)	67 (32.7)	4 (4.9)	23 (40.4)	40 (60.6)	
Van Xa (27.2 μg/L)	8 (3.9)	5 (6.1)	3 (5.3)	0	
Education level [n (%)]					**0.019**
Elementary school	15 (7.3)	2 (2.4)	3 (5.3)	10 (15.2)	
Secondary school	135 (65.9)	51 (62.2)	44 (77.2)	40 (60.6)	
Diploma	40 (19.5)	20 (24.4)	7 (12.3)	13 (19.7)	
College graduate	15 (7.3)	9 (11.0)	3 (5.3)	3 (4.5)	
Maternal occupation [n (%)]					0.074
Housewife	32 (15.6)	10 (12.2)	9 (15.8)	13 (19.7)	
Agricultural worker	91 (44.4)	31 (37.8)	26 (45.6)	34 (51.5)	
Factory worker	44 (21.5)	18 (22.0)	11 (19.3)	15 (22.7)	
Employee	30 (14.6)	18 (22.0)	10 (17.5)	2 (3.0)	
Vendor	8 (3.9)	5 (6.1)	1 (1.8)	2 (3.0)	
Parity [n (%)]					0.477
1 person	75 (36.6)	32 (39.0)	21 (36.8)	22 (33.3)	
2 persons	100 (48.8)	41 (50.0)	30 (52.6)	29 (43.9)	
3 persons	25 (12.2)	8 (9.8)	5 (8.8)	12 (18.2)	
≥ 4 persons	5 (2.4)	1 (1.2)	1 (1.8)	3 (4.5)	
Antenatal care services [n (%)]					0.464
Province clinic hospital	20 (9.8)	9 (11.0)	7 (12.3)	4 (6.1)	
District clinic hospital	23 (11.2)	11 (13.4)	8 (14.0)	4 (6.1)	
Health center of commune	21 (10.2)	9 (11.0)	7 (12.3)	5 (7.6)	
Private medical center	125 (61.0)	48 (58.5)	32 (56.1)	45 (68.2)	
Not having antenatal care	16 (7.8)	5 (6.1)	3 (5.3)	8 (12.1)	
Complications during pregnancy [n (%)]					0.683
No	189 (92.2)	74 (90.2)	53 (93.0)	62 (93.9)	
Yes	16 (7.8)	8 (9.8)	4 (7.0)	4 (6.0)	
History of miscarriage [n (%)]					0.409
Never	175 (85.4)	71 (86.6)	52 (91.2)	52 (78.8)	
1 time	23 (11.2)	7 (8.5)	5 (8.8)	11 (16.7)	
2 times	4 (2.0)	2 (2.4)	0	2 (3.0)	
≥ 3 times	3 (1.5)	2 (2.4)	0	1 (1.5)	
Urinary Cotinine (μg/mmol creatinine)					
Median (range)	nd (nd-8.03)	nd	nd (nd-8.03)	nd	
Infant birth outcomes					
Gender					0.220
Male [n (%)]	109 (53.7)	45 (54.9)	30 (53.6)	34 (52.3)	
Female [n (%)]	94 (46.3)	37 (45.1)	26 (46.4)	31 (47.7)	
Birth length (cm) (mean ± SD)	49.8 ± 2.2	50.4 ± 2.0	49.1 ± 2.8	49.8 ± 1.4	**0.001**
Birth weight (kg) (mean ± SD)	3.1 ± 0.8	3.2 ± 0.8	3.0 ± 0.8	3.0 ± 0.8	0.154
Head circumference (cm) (mean ± SD)	32.2 ± 2.7	31.9 ± 2.1	32.6 ± 4.0	32.2 ± 1.8	0.336

^a^Statistically significant difference among groups at *p* <  0.05 by One-way ANOVA are highlighted in bold

^b^Mean arsenic concentrations in drinking water obtained from Do et al., 2013 [5]

**Table 3:** DNA damage in cord blood of newborns exposed to arsenic *in utero*

Types of DNA damage	Maternal arsenic exposure by toenail As			FDR-adjusted *p*-value^a^
	Low (< 0.5 μg/g)	Medium (0.5-1 μg/g)	High (>1 μg/g)	
8-OHdG (ng/mL)	0.24±0.01^b^	0.26±0.01	0.28±0.01^**^	**0.010**
	0.24 (0.13-0.44)^c^	0.26 (0.18-0.49)	0.26 (0.19-0.55)	
	(n=81)	(n=52)	(n=61)	
8-Nitroguanine (ng/mL)	157.66±13.85	183.21±17.90	229.94±23.34^*^	**0.032**
	117.20 (13.59-426.51)	154.99 (22.51-540.60)	178.77 (26.73-831.84)	
	(n=78)	(n=53)	(n=58)	
DNA strand breaks				
Tail length (μm)	2.37±0.08	2.81±0.10^**^	3.04±0.09^***^	**0.000**
	2.38 (0.65-4.11)	2.74 (1.13-4.03)	2.98 (1.69-4.92)	
	(n=73)	(n=48)	(n=54)	
Olive tail moment (μm)^d^	0.20±0.01	0.24±0.01^**^	0.27±0.01^***^	**0.000**
	0.19 (0.07-0.39)	0.22 (0.07-0.44)	0.26 (0.11-0.49)	
	(n=73)	(n=48)	(n=54)	
%DNA in tail	1.36±0.06	1.62±0.08^*^	1.94±0.10^***, ##^	**0.000**
	1.28 (0.53-3.00)	1.51 (0.37-2.88)	1.82 (0.48-4.05)	
	(n=73)	(n=48)	(n=54)	

^a^The raw p-values were subjected to multiple testing correction using FDR-adjusted *p*-value; Statistically significant difference among groups at p <0.05 by One-way ANOVA are highlighted in bold

Values are expressed as mean ± SE (b) and Median (minimum - maximum) (c)

^*, **, ***^ Statistically significant difference from corresponding low- exposed group at *p* < 0.05, < 0.01 and < 0.001, respectively by Mann-Whitney U test

^##^ Statistically significant difference from corresponding medium-exposed group at *p*< 0.01 by Mann-Whitney U test

^d^ Olive tail moment is the product of the tail length and the fraction of total DNA in the tail.

**Table 5:** Univarite analysis of associations among the study parameters

	Coefficient β (95% CI)									
	DNA base damage		DNA strand breaks			Micronucleus frequency		Arsenic concentration		
	8-OHdG	8-nitro guanine	Tail length	Olive tail moment	%DNA in tail	Mono nucleated	Bi nucleated	Urine (μg/g creatinine)	Cord blood	Toenail
*Arsenic exposure*										
Maternal toenail	0.068^**^	0.244^**^	0.112^***^	0.083^*^	0.131^**^	0.698^**^	-0.045	0.225^***^	0.126^**^	
	(0.023,0.133)	(0.078,0.410)	(0.056,0.168)	(0.017,0.149)	(0.056,0.207)	(0.272,1.124)	(-0.167,0.077)	(0.113,0.336)	(0.043,0.209)	
Maternal urine										
Total As	0.003	-0.017	0.058	0.135^**^	0.100^*^	0.378	0.128			
	(-0.054,0.061)	(-0.229,0.196)	(-0.014,0.129)	(0.051,0.220)	(0.004,0.197)	(-0.166,0.922)	(-0.028,0.084)			
iAs	-0.021	0.155	-0.009	0.043	0.02	-0.085	0.051	-0.054	0.007	-0.184
	(-0.079,0.037)	(-0.060,0.371)	(-0.081,0.064)	(-0.043,0.129)	(-0.078,0.118)	(-0.637,0.468)	(-0.107,0.209)	(-0.205,0.097)	(-0.103,0.117)	(-0.375,0.007)
MMA	0.019	0.141	0.010	0.047	0.039	0.496	0.115	-0.107	0.054	0.215^*^
	(-0.039,0.077)	(-0.075,0.356)	(-0.063,0.082)	(-0.039,0.133)	(-0.059,0.137)	(-0.056,1.048)	(-0.043,0.273)	(-0.257,0.043)	(-0.056,0.163)	(0.025,0.405)
DMA	-0.01	-0.238	0.011	-0.057	-0.025	-0.146	-0.070	0.445^***^	-0.097	0.003
	(-0.075,0.055)	(-0.475,0.003)	(-0.070,0.092)	(-0.153,0.039)	(-0.135,0.084)	(-0.763,0.470)	(-0.246,0.106)	(0.290,0.600)	(-0.218,0.025)	(-0.212,0.218)
Cord blood	-0.056	-0.086	0.192^***^	0.256^***^	0.244^***^	0.456	0.774^***^	0.042		
	(-0.135,0.023)	(-0.379,0.206)	(0.094,0.290)	(0.140,0.373)	(0.111,0.377)	(-0.292,1.204)	(0.560,0.988)	(-0.163,0.246)		
*DNA damage*										
8-OHdG		0.025	0.082	-0.119	0.040	0.016	-0.035			
		(-0.021,0.071)	(-0.083,0.247)	(-0.356,0.119)	(-0.148,0.229)	(-0.007,0.038)	(-0.096,0.026)			
8-nitroguanine			0.043	0.170	0.459	-0.008	-0.063			
			(-0.563,0.649)	(-0.700,1.040)	(-0.226,1.144)	(-0.091,0.075)	(-0.286,0.160)			
*DNA strand breaks*										
Tail length				0.432^***^	0.063	0.002	0.064^*^			
				(0.201,0.663)	(-0.128,0.255)	(-0.021,0.026)	(0.003,0.125)			
Olive tail moment					0.574^***^	0.033^***^	0.008			
					(0.482,0.666)	(0.018,0.048)	(-0.035,0.051)			
%DNA in tail						0.003	0.019			
						(-0.018,0.023)	(-0.036,0.074)			

^*, **,***^Statistically significant association at *p* < 0.05, *p* < 0.01 and *p* < 0.001, respectively

β represents standardized coefficient; 95% CI represents 95% confidence interval (CI)

Olive tail moment is the product of the tail length and the fraction of total DNA in the tail

**Table 6:** Multivariate regression analyses between arsenic exposure and early genotoxic effects in newborns

	Coefficient β [adjusted p-value] (95%CI)						
	DNA base damage		DNA strand breaks			Micronucleus frequency	
	8-OHdG	8-Nitroguanine	Tail Length	Olive Mom.	%DNA in tail	Mononucleated	Binucleated
Toenail As	0.234 **[0.010]**	0.210 **[0.031]**	0.360 **[0.000]**	0.192 **[0.000]**	0.273 **[0.000]**	0.325 **[0.000]**	−0.029 [0.958]
(μg/L)	(0.089, 0.379)	(0.064, 0.356)	(0.221, 0.499)	(0.045, 0.339)	(0.124, 0.422)	(0.177, 0.472)	(−0.175, 0.118)
Urinary Total As	0.003 [0.974]	−0.063 [0.518]	0.142 [0.140]	0.232 **[0.000]**	0.165 [0.073]	0.100 [0.220]	0.090 [0.146]
(μg/g creatinine)	(−0.153, 0.158)	(− 0.219, 0.094)	(− 0.001, 0.285)	(0.089, 0.376)	(0.020, 0.310)	(−0.056, 0.256)	(− 0.052, 0.232)
Urinary iAs	−0.038 [0.974]	0.109 [0.423]	0.021 [0.781]	0.093 [0.112]	0.060 [0.359]	−0.034 [0.339]	0.050 [0.146]
(μg/L)	(−0.183, 0.107)	(− 0.037, 0.255)	(− 0.114, 0.156)	(− 0.042, 0.228)	(−0.077, 0.197)	(− 0.213, 0.144)	(− 0.119, 0.219)
Urinary MMA	0.012 [0.974]	0.076 [0.518]	0.054 [0.538]	0.087 **[0.027]**	0.073 [0.268]	0.163 [0.091]	0.153 [0.114]
(μg/L)	(−0.137, 0.161)	(−0.074, 0.226)	(− 0.085, 0.192)	(− 0.052, 0.226)	(−0.068, 0.214)	(0.016, 0.311)	(0.011, 0.295)
Urinary DMA	−0.006 [0.974]	−0.060 [0.518]	0.085 [0.538]	0.003 [0.056]	0.029 [0.375]	−0.012 [0.255]	− 0.011 [0.227]
(μg/L)	(−0.155, 0.143)	(− 0.209, − 0.090)	(−0.053, 0.223)	(− 0.148, 0.153)	(− 0.123, 0.181)	(− 0.208, 0.183)	(− 0.199, 0.177)
Cord blood	−0.105 [0.503]	− 0.026 [0.732]	0.270 [0.001]	0.316 **[0.000]**	0.264 **[0.001]**	0.094 [0.255]	0.519 **[0.000]**
(μg/L)	(−0.254, 0.044)	(−0.177, 0.125)	(0.131, 0.409)	(0.178, 0.454)	(0.125, 0.404)	(− 0.054, 0.242)	(0.380, 0.657)

Model was adjusted for the covariates including age, BMI, education, occupation, gestational age of sample collection during pregnancy (urine and nail samples) and baby delivery (cord blood sample)

The raw p-values were subjected to multiple testing corrections using FDR-adjusted *p*-value and the statistically significant are highlighted in bold

β represents standardized coefficient; 95% CI represents 95% confidence interval (CI)

Olive tail moment is the product of the tail length and the fraction of total DNA in the tail

The corrected Tables are as follows:

The original article has been corrected.
